# Analysis of a Urinary Biomarker Panel for Obstructive Nephropathy and Clinical Outcomes

**DOI:** 10.1371/journal.pone.0112865

**Published:** 2014-11-17

**Authors:** Yuanyuan Xie, Wei Xue, Xinghua Shao, Xiajing Che, Weijia Xu, Zhaohui Ni, Shan Mou

**Affiliations:** 1 Department of Nephrology, Molecular Cell Lab for Kidney Disease, Ren Ji Hospital, School of Medicine, Shanghai Jiao Tong University, Shanghai, P.R. China; 2 Department of Urology, Ren Ji Hospital, School of Medicine, Shanghai Jiao Tong University, Shanghai, P.R. China; INSERM, France

## Abstract

**Objectives:**

To follow up renal function changes in patients with obstructive nephropathy and to evaluate the predictive value of biomarker panel in renal prognosis.

**Methods:**

A total of 108 patients with obstructive nephropathy were enrolled in the study; 90 patients completed the follow-up. At multiple time points before and after obstruction resolution, urinary samples were prospectively collected in patients with obstructive nephropathy; the levels of urinary kidney injury molecule-1 (uKIM-1), liver-type fatty acid-binding protein (uL-FABP), and neutrophil gelatinase associated lipocalin (uNGAL) were determined by enzyme-linked immunosorbent assay (ELISA). After 1 year of follow-up, the predictive values of biomarker panel for determining the prognosis of obstructive nephropathy were evaluated.

**Results:**

uKIM-1 (*r* = 0.823), uL-FABP (*r* = 0.670), and uNGAL (r = 0.720) levels were positively correlated with the serum creatinine level (all *P*<0.01). The levels of uKIM-1, uL-FABP, and uNGAL were higher in the renal function deterioration group than in the renal function stable group. Cox regression analysis revealed that the 72-h postoperative uKIM-1 level and the preoperative and 72-h postoperative uL-FABP levels were all risk factors for renal function deterioration (all *P*<0.01). The area under the curve of Receiver Operating Characteristic(ROC-AUCs) of 72-h postoperative uKIM-1, preoperative uL-FABP, and 72-h postoperative uL-FABP were 0.786, 0.911, and 0.875, respectively. When the combined preoperative uKIM-1, uL-FABP, and uNGAL levels or combined 72-h postoperative uKIM-1, uL-FABP, and uNGAL levels were considered, the accuracy of prediction for renal prognosis was markedly increased, with an ROC-AUC of 0.967 or 0.964, respectively. Kaplan-Meier survival curve analysis demonstrated that a 72-h postoperative uKIM-1>96.69 pg/mg creatinine (Cr), a preoperative uL-FABP>154.62 ng/mg Cr, and a 72-h postoperative uL-FABP>99.86 ng/mg Cr were all positively correlated with poor prognosis (all *P*<0.01).

**Conclusion:**

Biomarker panel may be used as a marker for early screening of patients with obstructive nephropathy and for determining poor prognosis.

## Introduction

Obstructive nephropathy is a common cause of acute kidney injury (AKI), chronic kidney disease (CKD) and end-stage renal disease (ESRD) [1]–[4]. Nephrolithiasis is an independent, albeit small, risk factor for CKD. The renal function of patients with obstructive nephropathy is initially slowly decreased, followed by rapid deterioration of renal function once AKI occurs. Even during the early stage of AKI, the occurrence of AKI may facilitate the progression of the disease as well as earlier progression into ESRD [5]. Ten years after discharge from the hospital, 24.0% to 61.6% of AKI patients have progressed into stage 3 and stage 5 CKD, respectively [6]. As a severe complication, AKI is now recognized as a short- and long-term risk factor for poor prognosis. Several biomarkers for the early diagnosis of AKI have been proposed, including kidney injury molecule-1 (KIM-1), liver-type fatty acid-binding protein (L-FABP), and neutrophil gelatinase-associated lipocalin (NGAL) [7]. However, the usefulness of these markers for evaluating renal disease progression remains unclear [8]. Studies of the application of urinary KIM-1 (uKIM-1) and urinary L-FABP (uL-FABP) in obstructive nephropathy have been limited, and further studies are needed to validate their use as predictive parameters for the prognosis of obstructive nephropathy progression. In this study, uKIM-1 and uL-FABP levels were measured at multiple time points during long-term follow-up of patients with obstructive nephropathy to investigate the potential predictive values of these biomarkers as renal prognostic parameters in patients with obstructive nephropathy.

## Subjects and Methods

### Subjects

The criteria for inclusion in the study were as follows: patients with obstructive nephropathy confirmed by surgery performed at our institute, age ≥18 years old, male or female, with complete clinical records and diagnosis using urinary system ultrasound, CT scan, or kidney radioisotope scanning. The etiology for obstructive nephropathy in all patients was lithiasis. There were unilateral incomplete obstructions, unilateral complete obstructions and bilateral incomplete obstructions. The exclusion criteria were as follows: patients <18 years old, ESRD with regular hemodialysis or peritoneal dialysis, AKI of other causation, prior history of kidney diseases, immune system diseases, solid tumors or other complications, or clinical presentation indicating acute or chronic infection.

Obstructive nephropathy refers to the renal disease resulting from impaired flow of urine or tubular fluid as a consequence of structural or functional abnormalities in the urinary tract [9]. Obstructive nephropathies in all subjects were confirmed by imaging results and surgery. Functional impairment referred to split renal function decline in radionuclide scan.

The study was approved by the Ethics Committee of Ren Ji Hospital, School of Medicine, Shanghai Jiao Tong University, Shanghai, 200127, P.R. China and all participants gave written informed consent.

### Urine sample collection

A 10-mL sample of fresh urine was collected preoperatively and 4 h, 8 h, 12 h, 24 h, 48 h, and 72 h postoperatively, respectively, for each patient and centrifuged at 1,000×*g* for 15 min. The supernatant was transferred to an Eppendorf tube and stored at −80°C until use.

### Determination of biological parameters

Serum creatinine (sCr) and urinary creatinine (uCr) were determined using an enzyme assay. uKIM-1 levels were determined by enzyme-linked immunosorbent assay (ELISA) (R&D Company, Minneapolis, USA). The corresponding concentrations in the samples were calculated based on the standard curve and expressed as pg/mg Cr after synchronous correction with uCr. The uL-FABP level was determined by ELISA (Hycult Biotech, Uden, The Netherlands). The corresponding concentrations in the samples were calculated based on the standard curve and expressed as ng/mg Cr after synchronous correction with uCr. The uNGAL level was determined by ELISA (Hycult Biotech, Uden, The Netherlands). The corresponding concentrations in the samples were calculated based on the standard curve and expressed as ng/mg Cr after synchronous correction with uCr. The glomerular filtration rate (GFR) was estimated using the simplified formulation of modified diet in renal disease (MDRD), i.e., eGFR = 186×(sCr/88.4)^−1.154^×age^−0.203^×(0.742, female) [10].

### Grouping

After 1-year of follow-up, the patients were classified into two groups: the stable renal function group and the deteriorated renal function group. Stable renal function was defined as a sCr that decreased to the baseline level before obstruction, increased <50% versus the baseline level, or a stable sCr level within the normal range.

### Statistical considerations

Statistical analysis was performed using SPSS13.0 software. Normally distributed data of normal were expressed as *mean*±SD, and the *t* test was used for inter-group comparisons; non-normally distributed data were expressed using the median (M) and interquartile range (*P*_25_, *P*_75_), and the rank sum test was used for inter-group comparison. Spearman correlation analysis was used to analyze correlations. The sensitivity and specificity of uKIM-1 and uL-FABP for renal prognosis were evaluated using the ROC curve and area under the curve. Relevant risk factors that might influence renal prognosis were analyzed using Cox multifactor regression analysis. The Kaplan-Meier survival curve was used to analyze the survival rate. Statistical significance was set at *P*<0.05.

## Results

### General information

**Figure 1:**
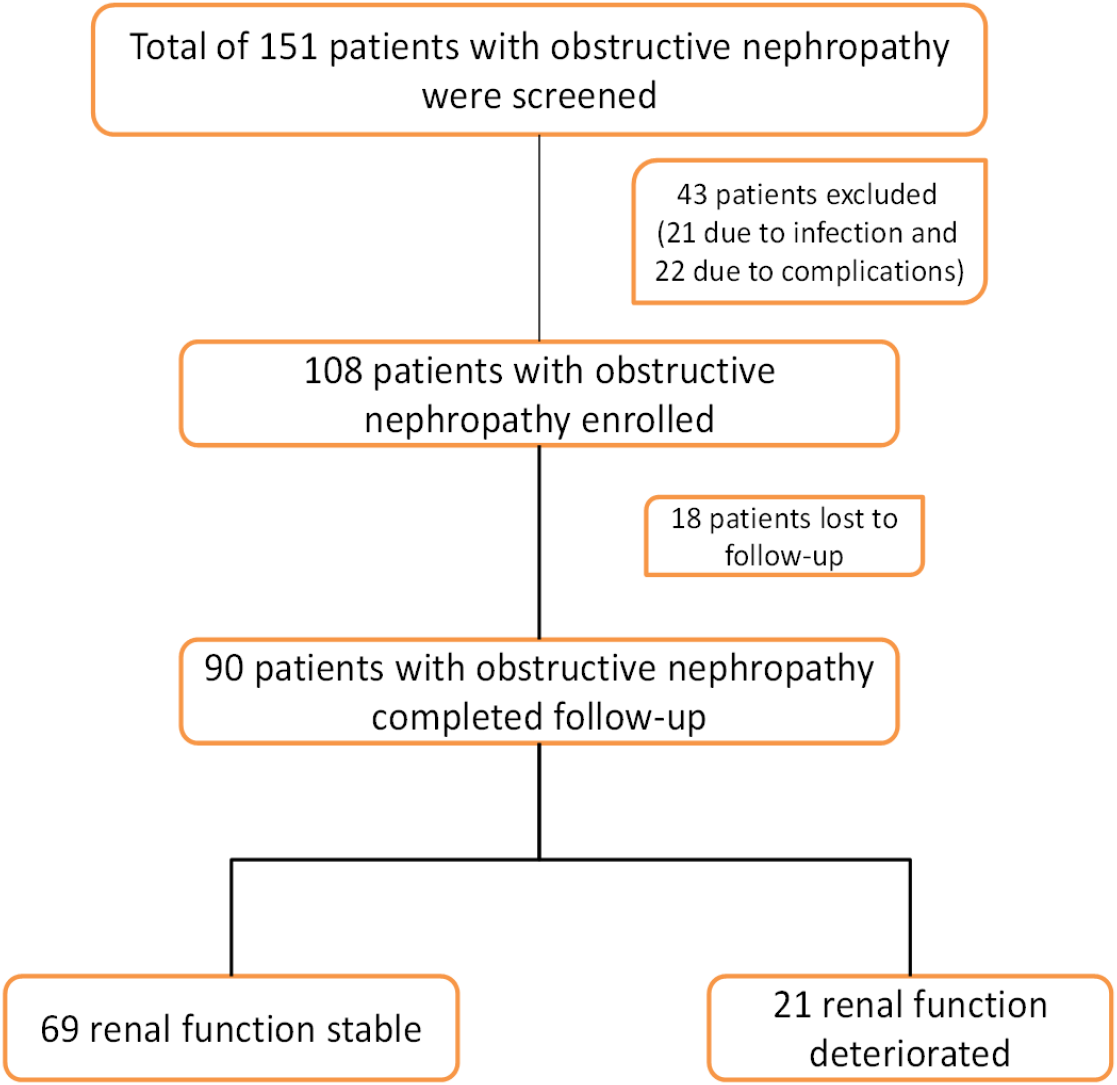
Study flow chart.

A total of 151 patients were screened, and 108 were enrolled in the study; 18 patients were lost to follow-up and excluded from the study. Ultimately, 90 patients with obstructive nephropathy were included in the study; of these, 54 patients progressed to AKI, and 36 did not progress to AKI. At 1-year follow-up, 69 patients were classified as renal function stable, and 21 were classified as renal function deteriorated (Figure 1).

The age of the 90 patients with obstructive nephropathy ranged from 37 to 76 years (average 54.69±9.87). The male/female ratio was 2.1∶1. The level of serum creatinine (sCr) was 454.64 (73.1–873.23) µmol/L, eGFR was 9.7 (5.2–93) mL/min/1.73 m^2^, uNAG was 20.53 (14.93–56.54) U/mg Cr, uKIM-1 was 433.23 (160.43–628.12) pg/mg Cr, uL-FABP was 130.13 (36.67–755.07) ng/mg Cr, and uNGAL was 386.03 (103.25–1286.75) ng/mg Cr. The uKIM-1, uNGAL, and uL-FABP levels were higher in patients with obstructive nephropathy than in the normal control group (Table 1).

**Table 1:** Characteristics of the study group and healthy subjects.

Variable	ON patients	Healthy subjects	P
**Sex (n; male/female)**	90 (61/29)	28 (16/12)	NS
**Age (years)**	54.69±9.87	50.77±6.25	NS
**Body weight (kg)**	69.56±11.68	68.11±10.91	NS
**RBC (×10^12^/L)**	4.15 (3.48–4.63)	5.10 (3.28–6.36)	NS
**WBC (×10^9^/L)**	6.79 (5.63–8.84)	5.43 (4.85–8.66)	NS
**Hemoglobin (g/dL)**	129.45 (104.25–140.5)	130.69 (105.1–140.5)	NS
**Serum albumin (g/L)**	43.41 (39.5–46.81)	42.03 (37.41–45.65)	NS
**ALT (IU/L)**	20.34 (7.35–25.35)	21.2 (7.55–25.15)	NS
**AST (U/L)**	17.65 (14.25–25.35)	16.5 (12.35–23.8)	NS
**Total cholesterol (mmol/L)**	5.3 (3.96–6.52)	5.60 (3.66–7.56)	NS
**Triglycerides (mmol/L)**	1.55 (1.39–2.98)	1.77 (1.43–2.82)	NS
**Renal function**			
** Serum creatinine (**µ**mol/L)**	454.64 (73.1–873.23)	71.96 (59.03–84.89)	0.001
** GFR (ml/min/1.73 m^2^)**	9.7 (5.2–93)	96.18 (78.22–109.9)	0.000
**Urinary NAG (U/mg)**	20.53 (14.93–56.54)	2.1 (1.6–10.5)	0.001
**Urinary L-FABP (ng/mg)**	130.13 (36.67–755.07)	20.12 (16.43–93.69)	0.001
**Urinary KIM-1 (pg/mg)**	433.23 (160.43–628.12)	109.03 (54.21–266.36)	0.002
**Urinary NGAL (ng/mg)**	386.03 (103.25–1286.75)	65.61 (39.9–263.25)	0.000

GFR, glomerular filtration rate; AST, glutamate oxaloacetate transaminase; ALT, glutamate pyruvate transaminase; L-FABP, liver-type fatty acid-binding protein; NGAL, neutrophil gelatinase-associated lipocalin; KIM-1, kidney injury molecule-1; NAG, N-acetyl-β-D-glucosaminidase; RBC, red blood cells; WBC, white blood cells.

### uKIM-1, uL-FABP, and uNGAL levels and correlation with sCr and eGFR

The Spearman correlation analysis demonstrated that the uKIM-1 level was positively correlated with the serum creatinine level (r = 0.823; P = 0.000) and negatively correlated with eGFR (r = −0.869; P = 0.000). The uL-FABP level was positively correlated with the serum creatinine level (r = 0.670; P = 0.000) and negatively correlated with eGFR (r = −0.681; P = 0.000). The uNGAL level was positively correlated with the serum creatinine level (r = 0.720; P = 0.003) and negatively correlated with eGFR (r = −0.784; P = 0.001).

### Comparison between the stable renal function group and the deteriorated renal function group

There were no significant differences in age, gender ratio, ALT, AST, total cholesterol (TC), triglycerides (TG), white blood cell count (WBC), red blood cell count (RBC), platelets (PLT), and uNAG levels between the stable renal function group and the deteriorated renal function group (all *P*>0.05). Hemoglobulin (Hb) and albumin (Alb) levels were higher in the deteriorated renal function group than the stable renal function group (all *P*<0.05).

**Figure 2:**
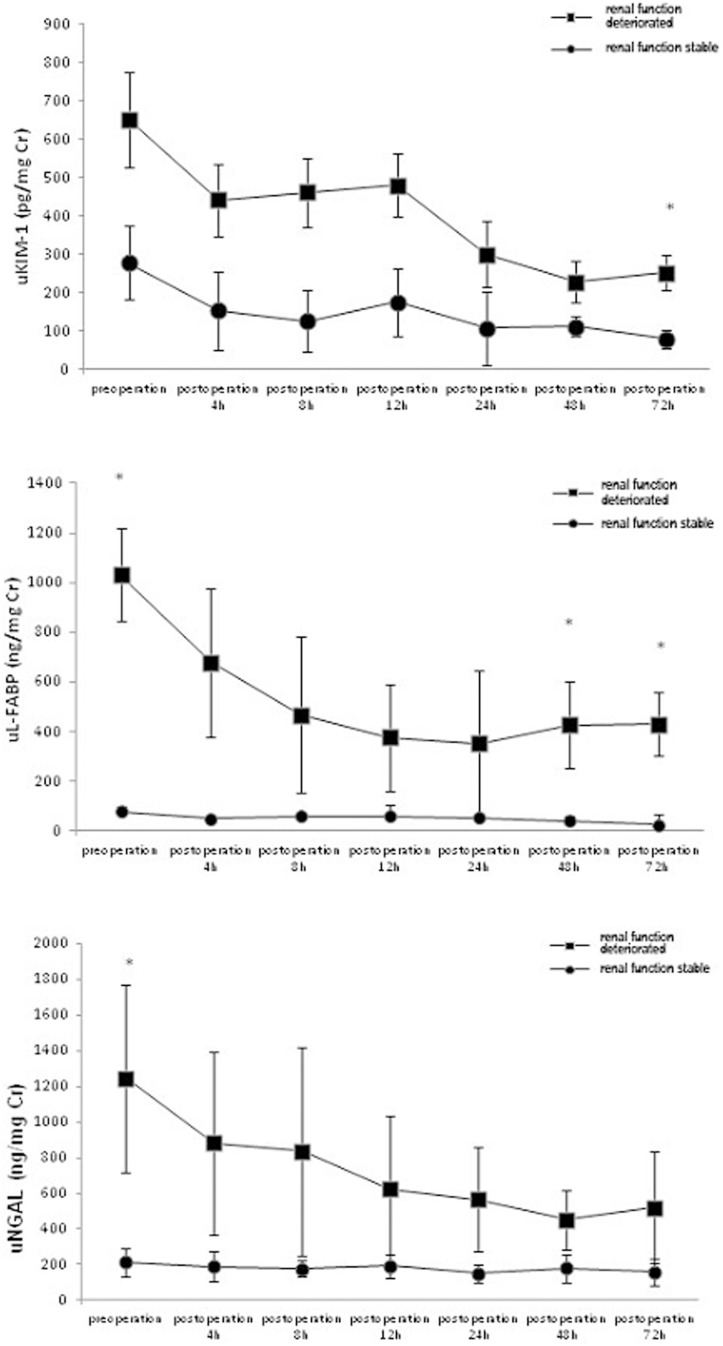
Comparison of the deteriorated renal function group and the stable group at 1-year follow-up. *The differences between the deteriorated renal function group and the stable group were statistically significant (P<0.05).

**Figure 3:**
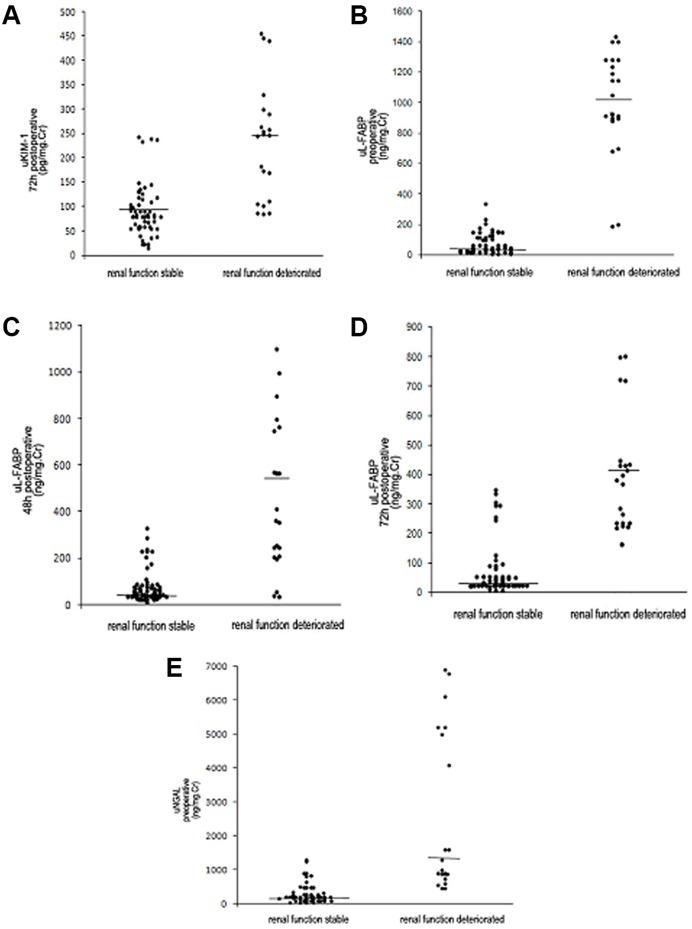
Distribution of biomarker levels in the deteriorated renal function group and the stable group at 1-year follow-up. A. Distribution of 72-h postoperative uKIM-1 levels in the deteriorated renal function group and the stable group at 1-year follow-up. B. Distribution of preoperative uL-FABP levels in the deteriorated renal function group and the stable group at 1-year follow-up. C. Distribution of 48-h postoperative uL-FABP levels in the deteriorated renal function group and the stable group at 1-year follow-up. D. Distribution of 72-h postoperative uL-FABP levels in the deteriorated renal function group and the stable group at 1-year follow-up. E. Distribution of preoperative uNGAL levels in the deteriorated renal function group and the stable group at 1-year follow-up.

uL-FABP, uKIM-1, uNGAL, and sCr levels were evaluated at various time points and compared between the stable renal function group and the deteriorated renal function group. The 48-h and 72-h postoperative uL-FABP levels were significantly higher in the deteriorated group than in the stable group. The 72-h postoperative uKIM-1 level was significantly higher in the stable group than in the deteriorated group. The preoperative uNGAL level was significantly higher in the deteriorated group than in the stable group. These differences were significant (all *P*<0.05) (Figure 2,3), whereas the sCr level was not statistically different between the two groups.

### Risk factor analysis of renal prognosis

Stepwise Cox regression analysis revealed that a 72-h postoperative uKIM-1 of 132.68±29.34 pg/mg Cr (*HR* = 1.008; 95% *CI,* 1.001–1.013); preoperative uL-FABP of 367.63±112.51 ng/mg Cr (*HR* = 1.002; 95% *CI,* 1.001–1.004); and 72-h postoperative uL-FABP of 220.36±59.65 ng/mg Cr (*HR* = 1.003; 95% *CI,* 1.001–1.007) were all risk factors for poor kidney prognosis (all *P*<0.01), whereas gender, age, and uNGAL level were not significantly correlated with prognosis (Table 2). Each 1 pg/mg drop in the72-h postoperative uKIM-1,1 ng/mg drop in the preoperative uL-FABP and 1 ng/mg drop in the 72-h postoperative uL-FABP was associated with a 0.8%, 0.2% and 0.3% increase in the risk of renal progression relatively.

**Table 2:** Relevant risk factors influencing the long-term renal prognosis of patients.

	Prognosis of patients		
	Mean ± standard deviation	HR (95% CI)	P
Preoperative uL-FABP (ng/mg Cr)	367.63±112.51	1.002 (1.001–1.004)	0.003
72 h postoperative uL-FABP (ng/mg Cr)	220.36±59.65	1.003 (1.001–1.007)	0.007
72 h postoperative uKIM-1 (pg/mg Cr)	132.68±29.34	1.008 (1.001–1.013)	0.009

### ROC curve analysis

**Figure 4:**
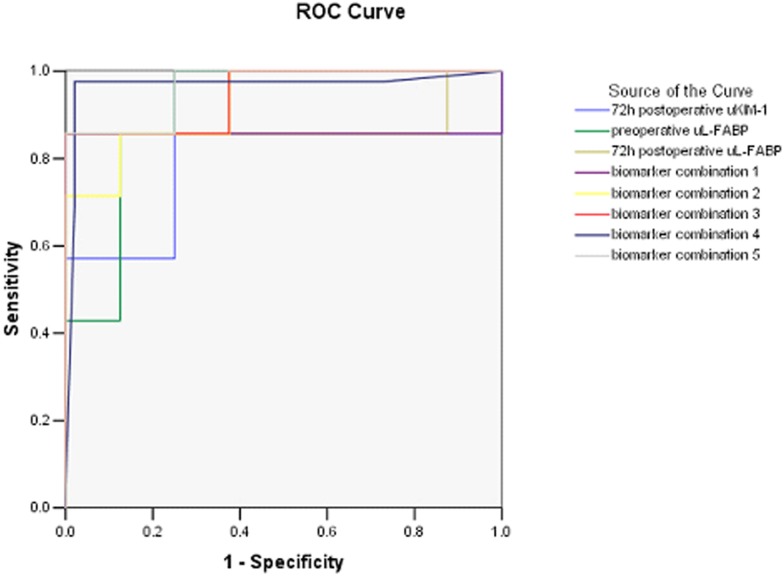
AUC for renal prognosis prediction. biomarker combination 1: combining preoperative uL-FABP and 72-h postoperative uL-FABP levels. biomarker combination 2: combining 72-h postoperative uL-FABP and uKIM-1 levels. biomarker combination 3: combining preoperative uL-FABP and uKIM-1 levels. biomarker combination 4: combining preoperative uL-FABP, uKIM-1, and uNGAL levels. biomarker combination 5: combining 72-h postoperative uL-FABP, uKIM-1, and uNGAL levels.

The area under the curve (AUC) of 72-h postoperative uKIM-1 was 0.786 (95% *CI*, 0.677–0.894; *P* = 0.008); when the intercept of detection was 96.69 pg/mg Cr, the sensitivity and specificity were 85.7% and 75%, respectively. The AUC of preoperative uL-FABP was 0.911 (95% *CI*, 0.851–0.971; *P* = 0.000); when the intercept of detection was 154.62 ng/mg Cr, the sensitivity and specificity were 85.7% and 87.5%, respectively. The AUC of 72-h postoperative uL-FABP was 0.875 (95% *CI*, 0.781–0.969, *P* = 0.000); when the intercept of detection was 99.86 ng/mg Cr, the sensitivity and specificity were 85.7% and 75%, respectively. When the preoperative uL-FABP and 72-h postoperative uL-FABP levels were substituted in the multifactor logistic regression model, the AUC of the combined biomarker was 0.857 (95% *CI*, 0.751–0.963, *P* = 0.001), with a sensitivity of 85.7% and a specificity of 87.5%. When the 72-h postoperative uL-FABP and uKIM-1 levels were substituted in the multifactor logistic regression model, the AUC of the combined biomarker was 0.929 (95% *CI*, 0.879–0.978; *P* = 0.000), with a sensitivity of 85.7% and a specificity of 87.5%. When the preoperative uL-FABP and uKIM-1 levels were substituted in the multifactor logistic regression model, the AUC of the combined biomarker was 0.946 (95% *CI*, 0.902–0.991; *P* = 0.000), with a sensitivity of 85.7% and a specificity of 100%. When the preoperative uL-FABP, uKIM-1, and uNGAL levels were substituted in the multifactor logistic regression model, the AUC of the combined biomarker was 0.967 (95%*CI*, 0.919–1.000; *P* = 0.000), with a sensitivity of 97.6% and a specificity of 97.9%. When the 72-h postoperative uL-FABP, uKIM-1, and uNGAL levels were substituted in the multifactor logistic regression model, the AUC of the combined biomarker was 0.964 (*CI*, 0.932–0.997; *P* = 0.000), with a sensitivity of 85.7% and a specificity of 100% (Figure 4, Table 3).

**Table 3:** AUC for renal prognosis prediction.

	ROC-AUC	Cut-off	Sensitivity	Specificity
72-h postoperative uKIM-1 (pg/mg Cr)	0.786 (0.677–0.894)	96.69	85.7%	75%
preoperative uL-FABP (ng/mg Cr)	0.911 (0.851–0.971)	154.62	85.7%	87.5%
72-h postoperative uL-FABP (ng/mg Cr)	0.875 (0.781–0.969)	99.86	85.7%	75%
preoperative uL-FABP+72-h postoperative uL-FABP	0.857 (0.751–0.963)		85.7%	87.5%
72-h postoperative uKIM-1+72-h postoperative uL-FABP	0.929 (0.879–0.978)		85.7%	87.5%
preoperative uL-FABP+preoperative uKIM-1	0.946 (0.902–0.991)		85.7%	100%
preoperative uL-FABP+preoperative uKIM-1+preoperative uNGAL	0.967(0.919–1.000)		97.6%	97.9%
72-h postoperative uL-FABP+72-h postoperative uKIM-1+72-h postoperative uNGAL	0.964 (0.932–0.997)		85.7%	100%

### Relationship between uKIM-1/uL-FABP and renal prognosis

**Figure 5:**
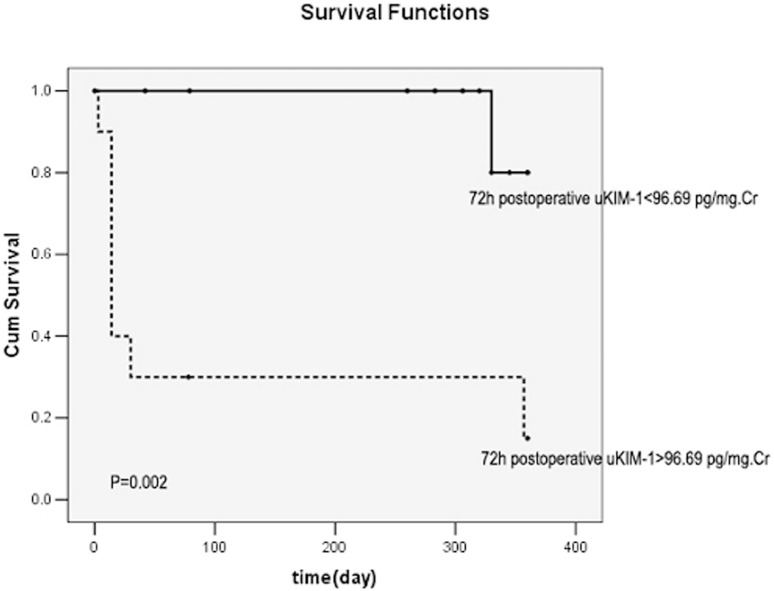
Relationship between the 72-h postoperative uKIM-1 level and renal prognosis. uKIM-1>96.69 pg/mg Cr was significantly correlated with poor prognosis.

**Figure 6:**
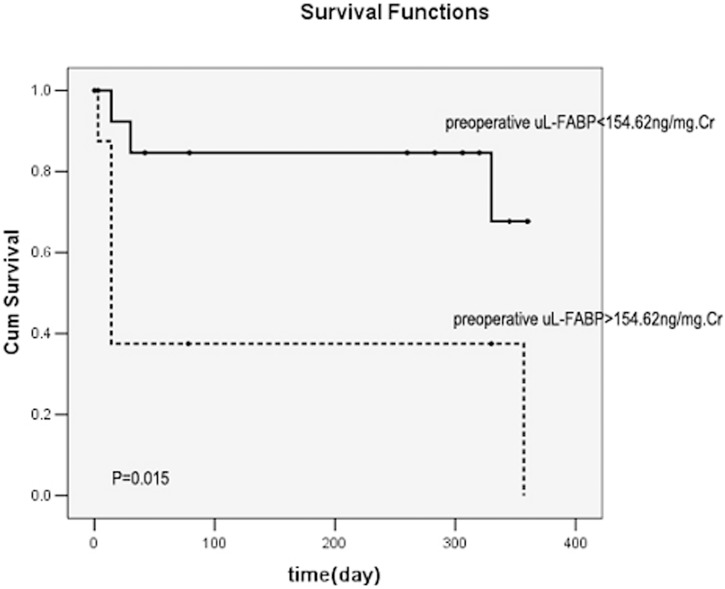
Relationship between the preoperative uL-FABP level and renal prognosis. uL-FABP>154.62 ng/mg Cr was significantly correlated with poor prognosis.

**Figure 7:**
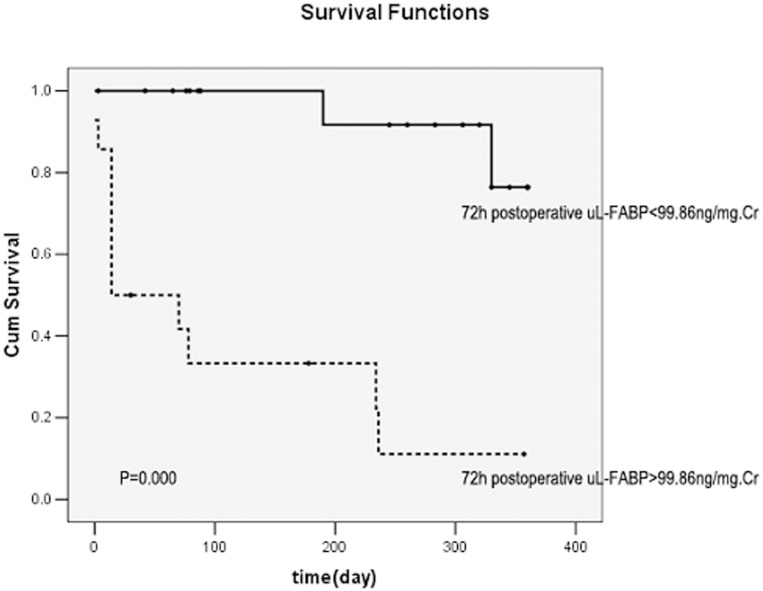
Relationship between the 72-h postoperative uL-FABP level and renal prognosis. uL-FABP>99.86 ng/mg Cr was significantly correlated with poor prognosis.

A 72-h postoperative uKIM-1>96.69 pg/mg Cr, preoperative uL-FABP>154.62 ng/mg Cr, and uL-FABP>99.86 ng/mg Cr were all positively correlated with poor kidney prognosis (all *P*<0.01) (Figures 5–7).

## Discussion

Obstructive nephropathy is a disease of renal functional and organic impairment that is caused by urine flow occlusion. Renal interstitial fibrosis is the characteristic pathological manifestation of obstructive nephropathy. Progressive renal interstitial fibrosis is directly related to renal function impairment and causes progressive renal function deterioration. Researchers and nephrologists aim to delay or block the deterioration of renal function and to establish parameters for prognosis prediction and early intervention to delay the progression of renal function deterioration [11]–[13].

KIM-1 is a transmembrane protein that is not expressed in normal kidneys but is secreted in large amounts by the renal proximal tubule cells in patients with renal diseases, renal ischemia, and renal toxic injury [14]. Pre-clinical experiments [15] have demonstrated that KIM-1 is an excellent marker for renal toxic injury. The uKIM-1 level has been correlated to various extents with tubular injury in 13 different AKI models, particularly mild tubular injury, in which uKIM-1 was more sensitive than sCr, blood urine nitrogen (BUN), and NAG. Chaturvedi et al [16] demonstrated that KIM-1 persists until the tubular cell injury is completely repaired. A large-scale clinical study [17] that examined renal needle biopsy samples from nephropathy patients with various etiologies demonstrated that proximal tubular cells from all patients secreted KIM-1 and that tissue KIM-1 expression was correlated with inflammation. uKIM-1 could reflect the KIM-1 level in tissues and correlate with renal interstitial inflammation and renal function. These factors indicated that KIM-1 is a biomarker for not only acute renal proximal tubular injury but also chronic renal interstitial inflammation and fibrosis. Our previous study [18] of AKI patients with obstructive nephropathy in our center showed that uKIM-1 could predict the renal outcome of AKI caused by obstructive nephropathy. In this study we found that uKIM-1 levels in patients with obstructive nephropathy were higher than healthy people. The results of this study demonstrated that uKIM-1 levels in patients with obstructive nephropathy were significantly different between the deteriorated renal function group and the stable renal function group; Cox regression analysis revealed that the 72-h postoperative uKIM-1 level after obstruction resolution was a risk factor for renal prognosis, with an AUC of 0.786; i.e., it could accurately predict renal function prognosis.

L-FABP is a small molecular protein that is secreted in large amounts at the site of active fatty acid metabolism and participates in and promotes the β-oxidation of fatty acids in the mitochondria or peroxisomes [19]. Under normal conditions, L-FABP exits the renal proximal tubular cells and participates in the metabolism of free fatty acids (FFAs) in renal tubules. By contrast, under various stress situations, the FFAs over-aggregate in the renal proximal tubules; the oxidated and peroxidated products may increase renal tubular injury, and L-FABP is expressed in the epithelial cells in large amounts [20]. In a clinical trial [21], uL-FABP levels were increased before angiography in 13 (high uL-FABP group) of 66 patients who underwent non-emergency angiography; during the follow-up period, these 13 patients were all diagnosed with contrast-associated nephropathy (CAN), whereas none of the patients with low uL-FABP levels (low uL-FABP group) were diagnosed with CAN. The pre-angiography sCr level was not significantly different between the two groups (*P*>0.05). These findings indicated that uL-FABP is a more sensitive biomarker than sCr for AKI prediction and may be used as a clinical parameter for the prediction of CAN. In a multicenter trial [22], 48 patients with non-diabetic nephropathy were retrospectively classified into a progression group and non-progression group and monitored for 7 to 13 months. The creatinine clearance rates (CCr) of the two groups were similar, whereas the uL-FABP level was significantly higher in the progression group than in the non-progression group (*P*<0.05). This finding indicated that L-FABP might predict the progression of chronic nephropathy. A study of chronic glomerulonephritis (CGN) conducted by our study group [23] demonstrated that uL-FABP levels were significantly higher in CGN patients than in the normal population. After 5 years of follow-up, uL-FABP levels were shown to be an important indicator of CGN progression; uL-FABP levels were significantly higher in the progression group than in the non-progression group. These findings indicated that uL-FABP is valuable for predicting CGN progression. Our previous study [24] of AKI caused by obstructive nephropathy had shown that uL-FABP was an early screening parameter for the poor prognosis of AKI patients with obstructive nephropathy. The uL-FABP levels were significantly higher in patients with obstructive nephropathy than in healthy people. All obstructive nephropathy patients had renal impairment, including the patients who did not develop AKI at the onset. The results of the present study demonstrated that there was a significant difference in uL-FABP levels between the deteriorated renal function group and the stable group and that the preoperative and 72-h postoperative uL-FABP levels are risk factors for renal prognosis that are positively correlated with poor prognosis. The AUC of preoperative uL-FABP was 0.911, and the sensitivity and specificity were 85.7% and 87.5%, respectively, when the detection intercept was 154.62 ng/mg Cr. The AUC of 72-h postoperative uL-FABP was 0.875, and the sensitivity and specificity were 85.7% and 75%, respectively, when the detection intercept was 99.86 ng/mg Cr. These findings all indicated that uL-FABP is an important marker of renal function deterioration in patients with obstructive nephropathy.

NGAL is a 25-kDa protein that is primarily secreted by neutrophils and various epithelial cells, including proximal tubular cells [25]. A meta-analysis [26] demonstrated that uNGAL was not only helpful for the early diagnosis of AKI but was also useful for predicting whether patients need RRT therapy and determining their short-term prognosis. In a large-scale community study of atherosclerosis risks [27], the uNGAL level was correlated with CKD stage 3 prediction and was a potential risk factor for predicting CKD. The predictive value of uNGAL for kidney function was not as high in our study of obstructive nephropathy.

Combining multiple biomarkers has recently been proposed to increase the accuracy of AKI prediction. Studies have demonstrated that combining markers with different sensitivities and specificities as well as combining different markers at various time points could increase the accuracy of the effective prediction of AKI [28], [29]. A prospective, multicenter cohort study that enrolled 1219 adults and 311 children demonstrated that there were several correlations between these biomarkers and that combining the uIL-18 and uL-FABP levels at various time points or combining the uIL-18, uNGAL. and uL-FABP levels at various time points increased the ROC-AUC of AKI prediction to 0.78. Because the etiology of AKI varies, combining different biomarkers was more beneficial for determining renal prognosis. A study [30] in 76 AKI patients receiving renal support therapy (RST) demonstrated that the maximal ROC-AUC of renal prognosis prediction was 0.94 when combining various biomarkers (uHGF, uNGAL, uCystatin C, uNGAL/MMP-9, and uIL-18) at multiple time points. The subjects in our study were patients with obstructive nephropathy, and when the various biomarker levels at different time points were substituted in the multifactor logistic regression model [31], the AUCs for determining obstructive nephropathy prognosis were 0.929 and 0.946, respectively, when combining biomarkers 72 h postoperative uKIM-1 and uL-FABP, preoperative uL-FABP and uKIM-1. The AUCs were 0.964 (sensitivity 85.7%, specificity 100%) and 0.967 (sensitivity 97.6%, specificity 97.9%), respectively, when combining biomarkers 72 h postoperative uKIM-1, uL-FABP and uNGAL, preoperative uKIM-1, uL-FABP and uNGAL. These results indicate that combining multiple biomarkers could markedly increase the accuracy of renal prognosis in patients with obstructive nephropathy.

This study is limited because it was a single-centered, small-sample clinical trial that followed renal function changes for 1 year without additional follow-up of biomarker level changes. Therefore, a large-scale, multicenter study is necessary to follow up the biomarker level changes during the progress of nephropathy and confirm the value of these markers in renal prognosis monitoring.

uKIM-1 and uL-FABP levels may accurately predict the renal prognosis of patients with obstructive nephropathy. The sensitivity and specificity of uL-FABP were superior to those of uKIM-1, which may be used as an early screening parameter for determining poor prognosis in patients with obstructive nephropathy. Combining various biomarkers may further increase the accuracy of renal prognosis in patients with obstructive nephropathy and thus help physicians give their patients timely intervention to prevent disease progression.
